# The response to COVID-19 in Argentina, Brazil, and Mexico: challenges to national coordination of health policies

**DOI:** 10.1590/0102-311XEN055023

**Published:** 2024-07-29

**Authors:** Cristiani Vieira Machado, Adelyne Maria Mendes Pereira, Carlos Machado de Freitas, Michele Souza e Souza, Sebastián Tobar, Suelen Carlos de Oliveira

**Affiliations:** 1 Escola Nacional de Saúde Pública Sergio Arouca, Fundação Oswaldo Cruz, Rio de Janeiro, Brasil.; 2 Instituto de Medicina Social, Universidade do Estado do Rio de Janeiro, Rio de Janeiro, Brasil.; 3 Centro de Relações Internacionais em Saúde, Fundação Oswaldo Cruz, Rio de Janeiro, Brasil.; 4 Universidade do Grande Rio, Mesquita, Brasil.

**Keywords:** Pandemics, COVID-19, Health Policies, Federalism, Pandemias, COVID-19, Políticas de Salud, Federalismo

## Abstract

The article analyzes the fight against COVID-19 in three Latin American countries: Argentina, Brazil, and Mexico. A multiple case study was carried out in a comparative perspective, based on a bibliographic review, documentary analysis, and secondary data, considering characteristics of the countries and the health system, evolution of COVID-19, national governance, containment and mitigation measures, health systems response, constraints, positive aspects and limits of responses. The three countries had distinct health systems but were marked by insufficient funding and inequalities when hit by the pandemic and recorded high-COVID-19 mortality. Structural, institutional, and political factors influenced national responses. In Argentina, national leadership and intergovernmental political agreements favored the initial adoption of centralized control measures, which were not sustained. In Brazil, there were limits in national coordination and leadership related to the President’s denialism and federative, political, and expert conflicts, despite a universal health system with intergovernmental commissions and participatory councils, which were little used during the pandemic. In Mexico, structural difficulties were associated with the Federal Government’s initial reluctance to adopt restrictive measures, limits on testing, and relative slowness in immunization. In conclusion, facing health emergencies requires strengthening public health systems associated with federative, intersectoral, and civil society coordination mechanisms and effective global solidarity mechanisms.

## Resumen

El artículo analiza la lucha contra el COVID-19 en tres federaciones latinoamericanas: Argentina, Brasil y México. Se realizó un estudio de casos múltiple en perspectiva comparada, basado en revisión bibliográfica, análisis documental y de datos secundarios, teniendo en cuenta: las características de los países y del sistema de salud, la evolución del COVID-19, la gobernanza nacional, las medidas de contención y mitigación, la respuesta de los sistemas de salud, los factores condicionantes, los aspectos positivos y los límites de las respuestas. Los tres países tenían sistemas de salud diferentes, pero marcados por financiación insuficiente y desigualdades, cuando afectados por la pandemia, y registraron una alta mortalidad por COVID-19. Las respuestas nacionales se influyeron por factores condicionantes estructurales, institucionales y políticos. En Argentina, el liderazgo nacional y los acuerdos políticos intergubernamentales favorecieron la adopción inicial de medidas de control centralizadas, que no se sustentaron. En Brasil, hubo límites en la coordinación y liderazgo nacional, relacionados con el negacionismo del presidente y los conflictos federativos, políticos y con expertos, a pesar de existir un sistema de salud universal que tiene comisiones intergubernamentales y consejos participativos, poco utilizados en la pandemia. En México, las dificultades estructurales se asociaron con la renuencia inicial del gobierno nacional en adoptar medidas restrictivas, límites en las pruebas y relativa lentitud en la vacunación. Se concluye que para enfrentar emergencias sanitarias hay que fortalecer los sistemas públicos de salud asociados con mecanismos de coordinación federativa, intersectorial y con la sociedad civil, así como mecanismos efectivos de solidaridad global.

## Introduction

National coordination of health policies involves challenges in federative countries, where the power of the State is shared by different spheres of government, involving disputes over power and resources ^1^. This is complex in large, heterogeneous, and unequal nations, such as the Latin American federations Argentina, Brazil, and Mexico.

These countries comprise 67% of the gross domestic product and 59% of the population of Latin America and the Caribbean ^2^, and have undergone transformations in State and society in recent decades, including democratization and political-administrative decentralization processes. Among the changes, health system reforms of different orientations stand out, with repercussions for the political-territorial organization, the population’s rights, and access to health ^3^.

As of March 2020, COVID-19 hit the Latin American region hard, which was experiencing an economic crisis, exacerbating inequalities in several dimensions ^4^. The pandemic revealed weaknesses in social and health policies in the countries, expressed in insufficient State capacity to deal with a complex health emergency, fragmentation of policies, and limits of communication with society.

Groups in situations of social vulnerability have suffered drastically from the economic, social, and health effects of the pandemic ^5^ due to precarious living, health, and employment conditions, aggravated by fragile social protection systems and insufficient investments in the public health system.

Fighting COVID-19 required articulating strategies such as physical distancing measures, namely isolation or quarantine, regulation of public spaces, individual and collective protection actions, health system reorganization, economic and social protection measures, and initiatives aimed at different territories and social groups. The countries’ responses varied in what regards adopting containment and mitigation measures and the capacity for coordination between spheres of government, public policies, and society, influencing the actions effectiveness. In federations, such processes were shaped by the political-territorial configuration of the State, power, and responsibilities of government spheres, characteristics of decentralization, and federative coordination mechanisms, in general, and in health ^6^ ^,^ ^7^.

The study of the three Latin American federations - Argentina, Brazil, and Mexico - is relevant, given the magnitude of the effects of COVID-19 in these countries, which represent 4.9% of the world population but accounted for 7% of cases and 17% of confirmed COVID-19 deaths in the world by December 2022 ^8^. In addition to combining, in a contradictory way, regional economic relevance and marked inequalities, these nations face challenges in coordinating policies in federative scenarios marked by institutional fragmentation and political conflicts.

Recognizing that the coordination of the response to health crises in federations holds specificities ^6^ ^,^ ^7^ ^,^ ^9^, the study aimed to identify the main characteristics, constraints, positive elements, and limits in the responses to the COVID-19 pandemic in these Latin American nations. From its results, we seek to extract lessons about the challenges of public health systems in facing health emergencies in countries marked by inequalities and difficulties in coordinating public policies.

## Methodology

A multiple case study was conducted in a comparative perspective, based on contributions from the historical-comparative approach of Social Sciences ^10^ and comparative literature on health systems ^11^. The available bibliography on the response and resilience of health systems in the face of COVID-19 ^12^ ^,^ ^13^ was also considered to identify the relevant analytical dimensions.

The selected countries were Argentina, Brazil, and Mexico, which are extensive, populous, and unequal federations. The study focused on the national policies to fight COVID-19, with an emphasis on coordinating strategies, considering the axes of analysis:

Context and characteristics of countries and the health system: socioeconomic, demographic, and health indicators, and configuration of the health system;

Epidemiological situation of COVID-19: evolution of incidence and mortality;

National governance: considering governance as a pattern of relationships between State and non-State actors, formal and informal, in institutional environments ^14^, we analyzed coordination between governments, policy areas and with other actors; leadership and communication with society;

Containment and mitigation: border control, physical distancing, regulation of commercial and leisure activities, economic measures, social and employment protection;

Health systems’ response to COVID-19 in surveillance and healthcare: information, active surveillance, testing, coordination between actions and services from primary to hospital care, immunization;

Constraints, positive aspects, and limits of countries’ responses.

The study was based on secondary sources. The characteristics of the countries and health systems were described with data from the Economic Commission for Latin America and the Caribbean (ECLAC) ^2^, World Bank ^15^, World Health Organization (WHO) ^16^, and Organization for Economic Cooperation and Development (OECD) ^17^, bases selected for their reliability and availability of international data, complemented by national data ^18^ ^,^ ^19^ ^,^ ^20^ ^,^ ^21^ ^,^ ^22^. The evolution of COVID-19, testing, and vaccination was described using data from Our World in Data database ^8^ and the Pan American Health Organization (PAHO) ^23^. Given the recent and dynamic nature of the pandemic, in addition to bibliographic review, research techniques involved analysis of government websites and documents (plans, communications, reports, notes, and minutes), notes from scientific and professional societies, and statements by public authorities, in articles or videos to characterize national responses.

The cases were characterized and analyzed based on a comparative perspective, looking for similarities and differences in the different axes, in the policy constraints, positive aspects, and limits of each country’s response to COVID-19.

## Results

### Context of countries and characteristics of health systems

The three Latin American federations are populous upper-middle-income countries with regional economic importance. The national processes of industrial modernization in the 20th century did not alter their peripheral insertion on the world stage, nor did they change structural inequalities evident since the colonial period. Poverty, income inequalities, and informality in the labor market are more pronounced in Brazil and Mexico than in Argentina. While life expectancy and the proportion of older people are higher in Argentina, the mortality rate from noncommunicable diseases and the prevalence of diabetes are higher in Brazil and Mexico, contributing to a high burden of disease. Regarding the structure of the health system, Mexico and Argentina have the largest rates of physicians, and Brazil has the largest rate of nurses to population. Argentina has the most significant availability of hospital beds, while Mexico has the lowest rates of hospital beds, as shown in Table 1.

**Table 1:** Demographic, socioeconomic, health, structure, and financing characteristics and health system coverage before the COVID-19 pandemic. Argentina, Brazil, and Mexico - 2019 or last available.

Characteristics	Argentina	Brazil	Mexico
Demographic			
Total population (in million) (2019) *	44,938	211,782	125,085
Population aged 65 and over (% of total population) (2019) *	11.6	9.0	7.9
Socioeconomic			
GDP per capita (USD) (2019) *	9,963.67	8,845.32	10,145.15
Poverty (% of population) (2019) **	11.2	26.2	31.1
Ratio of average family income per capita (quintile 5/quintile 1) (2019) **	8.5	19.1	11.9
Gini index (2019) **	0.429	0.535	0.467
Vulnerable jobs (% of total jobs) (2019) *	22.7	28.3	27.2
Health			
Life expectancy (years) (2019) ***	76.6	75.9	76.0
Standardized mortality rate from noncommunicable diseases (per 100,000 inhabitants) (2019) ***	435.2	424.5	464.8
Probability of death between 30-70 years due to CVD, cancer, diabetes, or CKD (2019) **	15.7	15.5	15.6
Prevalence of obesity among adults (BMI ≥ 30, % estimate) (2016) ***	28.5	22.3	28.4
Prevalence of diabetes (% of population aged 20-79) (2019) **	5.4	8.8	16.9
Health system structure			
Physicians (per 10,000 inhabitants) (2019) ***	39.8	23.0	24.7
Nurses (per 10,000 inhabitants) (2019) ***	25.9	73.7	28.8
Beds (per 10,000 inhabitants) (2017) ***	49.9	20.9	9.9
Intensive care beds (per 100,000 inhabitants) ^#^	19.0	21.6	3.3
Health system financing			
Health expenditure (% of GDP) (2019) ***	10.0	9.6	5.4
Government health expenditure (% of GDP) (2019) ***	6.1	3.9	2.7
Government health expenditure (% of general government expenditure) (2019) ***	1.399	599	542
Public health expenditure (% of total public expenditure) (2019) ***	16.1	9.2	10.3
Private health expenditure (% of health expenditure) (2019) ***	39.2	59.1	50.8
Out-of-pocket expenditure (% of health expenditure) (2019) ***	23.9	24.9	42.3
System coverage (per type)			
Public system (noncontributory and free access through state or contracted services) ^##^	100.0/34.8	100.0	36.5
Social security (contributory, for workers) ^##^	62.7	NA	61.1
Private sector of health plans and insurance/“prepaid” medicine ^##^	13.6	24.2	2.8

BMI: body mass index; CKD: chronic kidney disease; CVD: cardiovascular disease; GDP: gross domestic product; NA: not applicable; PPP: purchasing power parity.

Source: prepared by the authors, with data available in the databases:

* World Bank ^15^;

** Economic Commission for Latin America and the Caribbean ^2^. Argentina’s poverty, income ratio, and Gini data are only for the urban population, and those from Mexico refer to 2018;

*** World Health Organziation ^16^. Data from Argentina regarding nurses are from 2017;

^#^ For Argentina, 2019 data from the Argentine Health Information Integrated System, obtained from Gilardino et al. ^18^; for Brazil, data from January 2020 from the Brazilian Critical Care Association ^19^; for Mexico, 2017 data from Organization for Economic Cooperation and Development/World Bank ^17^;

^##^ For Argentina, 2019 data from the Argentine Ministryof Health, available in Tobar ^20^; the entire population can access public services (100%), and 34.8% only have access to them. For Brazil, whose system is public and universal (the Brazilian Unified National Health System), data on health plans and insurance from the Brazilian National Supplementary Health Agency ^21^, referring to 2019. For Mexico, the 2020 “*derechohabiencia*” data was used, from the Mexican National Institute of Statistics and Geography ^22^.

In the three countries, the organization of health policies in the first half of the 20th century occurred, on the one hand, through public health actions aimed at controlling infectious diseases, and on the other hand, by medical care for formal workers, in the segmented logic of social insurance. However, expanding coverage and configuration of health systems varied over time, and sectoral reforms from the 1980s onwards had different meanings ^3^ ^,^ ^24^.

In Argentina, the social insurance model was maintained through *Obras Sociales*, which are organizations linked to unions responsible for resource management and health care, initially by categories. The reforms of the 1980s and 1990s introduced market mechanisms that weakened corporate bases, such as the possibility of freely choosing *Obras Sociales* by workers and hiring health companies to provide services. This favored the expansion of the prepaid medicine sector, regulated by law from 2011. The public health subsystem, from primary to hospital care, is the responsibility of provinces and municipalities, with different configurations. Regarding legislation, organization, and inspection of services, the provinces are largely autonomous, under a decentralized supervision and financing by the Argentinian Ministry of Health. The Federal Health Council (COFESA, acronym in Spanish) is an instance of articulation between federal and provincial health authorities, whose institutionality and relevance varied over time ^25^. The difficulties of federative coordination in health express characteristics of Argentine federalism ^26^. The national health system is highly segmented (by groups of beneficiaries) and fragmented (organizationally) due to the nature of the *Obras Sociales*, provincial, and private sector subsystems, with complex interconnections between them ^27^.

In Brazil, the health reform of the 1980s led to the creation of the Brazilian Unified National Health System (SUS, acronym in Portuguese) in 1988, public and universal, whose implementation favored an increase in supply, coverage, and access to public services. There were transformations in the health care model, such as strengthening comprehensive policies and expanding primary care through the Family Health Strategy. Faced with political-administrative decentralization, emphasizing municipalities, the Brazilian Ministry of Health maintained regulatory power through federal regulations and financial transfers. The changes included arrangements for social participation and federative coordination in health, such as national and state intergovernmental committees, which gave institutionality to shared processes of formulation and monitoring of policies ^26^ ^,^ ^28^. As limits, notably, the insufficiency of health financing and the persistence of the private sector, subsidized by the State (since the 1960s), expressed in low public spending and a high proportion of private health spending, contradictory to the SUS ^29^.

In Mexico, in the 1980s and 1990s, the health system changed amid State reforms that emphasized economic liberalization, privatization, reduction of public spending, and decentralization ^30^. Pension reforms occurred and attempts to restrict medical care linked to the Mexican Social Security Institute (IMSS, acronym in Spanish) and the Institute for Social Security and Services for Civil Servants (ISSSTE, acronym in Spanish) were obstructed by the unions. From the 2000s onwards, the Popular Health Insurance (SPS, acronym in Spanish) was implemented, aimed at people with low incomes not covered by Social Security, who accessed decentralized public health services. In subsequent years, there was an increase in the population registered by the SPS, subject to controversy due to the limited nature of the services included in the basic package and the fact that registration does not guarantee access ^31^. In 2018, the new government abolished the SPS and created the Mexican Institute of Health for Welfare, initially implemented when COVID-19 hit the country. Characteristics of the Mexican system are organizational fragmentation, coverage segmentation, low public spending, and instability related to successive reforms.

Despite the differences between countries, before the COVID-19 pandemic, they all faced social problems such as poverty, income inequalities, and high labor informality, making it challenging to face the crisis. Their health systems were characterized by contradictions and limited actions, such as insufficient public spending and a high proportion of private spending on health, in the case of Brazil, mainly through private health insurance (despite the SUS), and in Mexico, through direct payment from families (Table 1).

### Epidemiological situation of COVID-19

The three countries were hit hard by COVID-19 from 2020 onwards, reaching high rates of cumulative mortality by the end of 2022 compared with other federations worldwide. Table 2 shows that, in the period, Brazil had the highest mortality rate, and Mexico had the highest estimates of lethality and excess mortality (concerning the historical series of deaths from all causes in previous years) compared with other federations worldwide. The incidence in the three countries is possibly underestimated due to low testing, which can lead to underreporting of cases and, to a lesser extent, deaths, affecting lethality.

**Table 2:** Indicators related to COVID-19 in Argentina, Brazil, Mexico, and other selected federations - until 2022.

Country	Cumulative incidence *	Cumulative mortality **	Excess mortality (%) ***	Lethality (%) ^#^			Tests (per 1,000 inhabitants) ^##^	Vaccination coverage (%) ^###^	Booster doses (per 100 inhabitants) ^§^
				2020	2021	2022			
Argentina	217,338.36	2,859.22	19.24	2.92	2.12	1.32	773.09	76.10	60.45
Brazil	167,775.53	3,217.37	20.44	2.56	2.78	1.92	330.91	78.29	48.22
Mexico	56,826.32	2,598.52	29.53	9.90	7.58	4.57	117.38	62.70	41.69
Canada	116,407.83	1,259.18	6.31	2.73	1.52	1.08	1,538.60	81.77	57.16
United States	292,706.07	3,192.46	14.04	1.81	1.57	1.09	2,483.63	67.28	37.64
Germany	446,707.54	1,987.98	6.32	2.83	1.68	0.45	1,357.56	76.03	68.33
South Africa	67,590.07	1,712.49	17.97	2.67	2.66	2.53	393.41	31.76	5.71
India	31,525.51	374.47	NA	1.45	1.38	1.19	551.94	64.19	2.95
Russia	150,395.92	2,718.36	24.29	1.80	2.93	1.81	1,928.73	51.36	10.09
Australia	408,521.74	680.59	4.45	3.25	0.65	0.17	2,487.17	82.68	61.53

NA: not available.

Source: Mathieu et al. ^8^.

* Cumulative incidence: confirmed cases/million inhabitants until 25/Dec/2022;

** Cumulative mortality: confirmed deaths/million inhabitants until 25/Dec/2022;

*** Excess mortality (%): the percentage difference between the accumulated deaths from all causes since January 2020 and the number of deaths projected for the period, based on previous years. Data for December 2022 for all countries except Argentina, which data is up to December 2021;

^#^ Lethality (%): ratio between deaths confirmed by COVID-19 and confirmed cases of COVID-19. The indicator is not an accurate measure of the risk of death, as the underreporting of mild cases hampers it. For each year, the last available data were obtained in the last week of December;

^##^ Tests: cumulative number of tests per 1,000 inhabitants, with variations in the unit of measurement: tests performed (Argentina, Brazil, Canada, United States, Germany, Russia, and Australia); people tested (Mexico and South Africa); samples tested (India). Data from 06/Mar/2022 for Germany and from 11/Mar/2022 for the other countries;

^###^ Vaccination coverage: % of the population with a complete initial vaccination schedule. Data are from 16/Jun/2022 for Australia and 24/Jun/2022 for the others. June was chosen for comparability purposes;

^§^ Booster doses: number of booster doses per 100 inhabitants. Data from 24/Jun/2022.

**Figure 1:**
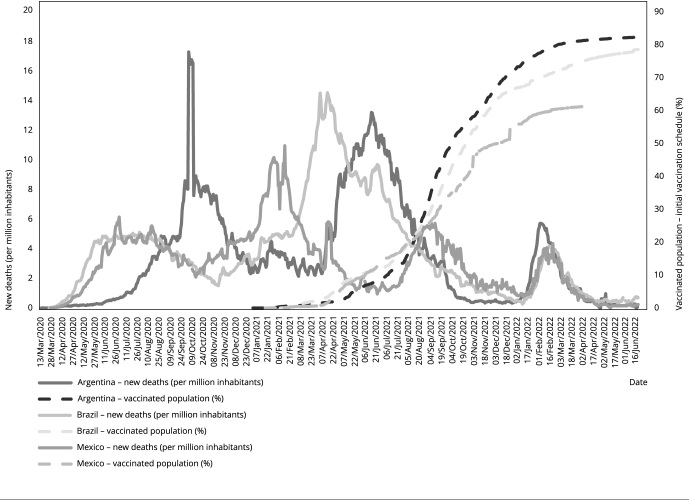
Evolution of new daily deaths from COVID-19 per million inhabitants and percentage of the population with a complete initial vaccination schedule. Argentina, Brazil, and Mexico. March 2020/June 2022.

Source: prepared by the authors. Data obtained from Mathieu et al. ^8^.

Figure 1 shows successive waves of mortality in the countries studied. A later and slower start to the first wave was observed in Argentina in 2020, favored by strict social distancing and surveillance measures in the first year of the pandemic, coordinated by the Federal Government in conjunction with the provinces. Subsequently, “social fatigue” led to the relaxation of measures ^20^ and a sharp increase in mortality, also influenced by the circulation of different virus variants.

The second wave began first in Mexico, but Brazil and Argentina reached high peaks in COVID-19 mortality. In the Brazilian case, this high wave lasted from the end of 2020 to mid-2021. Vaccination began gradually at the beginning of 2021. In the middle of the year, a more striking effect of the increase in vaccination coverage was observed in controlling mortality in Argentina and Brazil. A third wave occurred later that year in Mexico, which took longer to immunize the population. At the beginning of 2022, countries experienced a new wave, this time related to the Omicron variant, which led to very high number of cases, with lower mortality than that associated with previous waves, due to collective protection related to the advancement of vaccination.

### National governance, containment measures, and health systems response

National governance and response capacity to COVID-19 varied between countries, influenced by different federal arrangements and government positions regarding political orientation, leadership, and ability to dialogue with experts and other social groups.

Argentina has the most decentralized federative arrangement of the studied countries, with high autonomy for the provinces in health. In 2020, measures were centralized by the Federal Government, and federal coordination mechanisms involved meetings and agreements between political and health authorities. This articulation was essential for the initial control of COVID-19, including legal restrictions on mobility and economic and social activities to ensure physical distancing. Investments were mainly made in hospitals, expanding the number of beds.

Faced with the prolongation of the COVID-19 crisis, coordination strategies have weakened, given the very decentralized nature of Argentina, political competition, and the scarcity of more solid institutional arrangements for intergovernmental cooperation. In society, in a scenario of economic instability, resistance to containment measures grew, and “social fatigue” led to a reduction in the population’s adherence to physical distancing. The early start of immunization by December 2020 and its expansion in 2021, through purchases from different suppliers, was crucial to contain serious cases, hospitalizations, and deaths from COVID-19. There was also a regional production arrangement in partnership with Mexico, which had delays and limited results. The national research capacity, the effort of universities, and public-private partnerships allowed the local production and supply of masks, respirators, and other strategic inputs with federal induction and support ^32^.

Brazil is characterized by political-administrative decentralization of the health system with an emphasis on municipalities, but with the Federal Government’s power in inducing policies and a varied role for the states. Intergovernmental health committees at the national and state levels are essential for federative coordination in the SUS ^26^, and the role of participatory health councils in the three spheres of government is relevant for social control. However, these channels were not valued in the response to COVID-19, resulting in intergovernmental conflicts and coordination limits between governments and society. This was aggravated by the President of the Republic’s position of denialism, recommendation of measures lacking scientific evidence, and exacerbation of conflicts with opponents. Creating a national emergency committee was not enough to compensate for the problems of national leadership and coordination. There was a weakening of the Brazilian Ministry of Health as a national health authority, with three changes of ministers between 2020 and 2022 and delays in implementing relevant measures.

In a government marked by restrictions on federal public spending and setbacks in the social area, limited intersectoral coordination hampered the articulation between health, social protection, and economic policies to deal with the multiple dimensions of the crisis. As positive aspects, we highlight the existence of the SUS and an extensive primary health care network, whose potential to contribute to facing emergencies was little valued ^33^. Immunization highlighted contradictions in national policy. On the one hand, the country had a comprehensive National Immunization Program (PNI, acronym in Portuguese), and the national capacity to produce vaccines by two public institutions - Oswaldo Cruz Foundation (Fiocruz, acronym in Portuguese) (federal) and Butantan Institute (state) - that provided more than half of the doses administered until January 2023 ^23^ - including technology transfer agreements for national vaccine production. On the other hand, the Presidency’s position negatively affected immunization, with delays in buying supplies and vaccines and damaging statements about vaccines, weakening the PNI, which enjoyed high international credibility and legitimacy among the population.

In Mexico, the most centralized federation of the three, the leadership and coordination of the Federal Government would be decisive for the response, given low public spending, the fragmentation of the health system, and the ongoing health system reform process. However, initial hesitation in adopting the necessary containment measures hampered control of the spread of COVID-19. The organization of the response was based on previous experiences structuring health surveillance to deal with health emergencies. However, testing limits and slow vaccinations, which depended on acquisition from different suppliers, were weaknesses. Such limits, associated with the segmentation of the health system (expressed in the differences between subsystems regarding the development of guidelines, protocols, infrastructure, and inputs), persistent public underfunding, and structural inequalities in health conditions and access, contributed to high mortality by COVID-19 and excess mortality in the period.

Box 1 summarizes the characteristics of the three countries’ responses to COVID-19.

**Box 1:** Main characteristics of countries’ response to COVID-19. Argentina, Brazil, and Mexico, 2020 to 2022.

COUNTRY	MAIN CHARACTERISTICS OF COUNTRIES’ RESPONSE
Argentina	Governance and national coordination: suprasectoral governance efforts between government areas and dialogue with experts; agreed mechanisms of federative coordination in health, but with fragile institutionality; Containment and mitigation measures: initially, border closure strategies (with difficulties), mandatory distancing strategies, and economic and social protection measures. Then, “social fatigue” and resistance to measures - new waves; Health system response: segmented health system with robust hospital provision. Federal Government investments (increase in ICU beds, respirators, hiring). Innovations and search for coordination between sectors. Limited role of primary health care. Vaccination began in December 2020, and good availability of vaccines through purchase from various suppliers. Relevance of achieving high vaccination coverage from 2021 to contain serious cases and mortality.
Brazil	Governance and national coordination: limits in national leadership and federative and intersectoral coordination; scant consideration of scientific evidence and societal participation; Containment and mitigation measures: initially, border closure strategies (with difficulties), tensions between the Federal Government and experts, states and municipalities created their own regulation regarding distancing measures, and insufficient economic and social protection measures; Health system response: relevance of the SUS, but difficulties due to inequalities and low investments; emphasis on hospital care (expansion of beds), limited coordination between public and private sectors, little appreciation of primary health care in the response; in 2020, little testing, difficulties in importing inputs and equipment. Relevance of national production of tests and vaccines (responsible for more than half of the doses administered until 2022) with technology transfer. Decisive role of vaccination from mid-2021, with good vaccination coverage of adults with the primary initial schedule. However, there are difficulties in high coverage of booster doses among children.
Mexico	Governance and national coordination: effort on national guidelines, especially for surveillance, but difficulties in federal coordination with the states; Containment and mitigation measures: initial delay in adopting physical distancing measures; then national program, limited scope. Insufficiency and low coordination of measures to protect the economy, employment, and social protection; Health system response: segmented and fragmented health system (under reform), with infrastructure inequalities, limits on public financing, and availability of equipment and supplies. Surveillance strategies based on previous experience with health emergencies. Emphasis on hospital care and testing restricted to serious cases. Dependence on purchasing vaccines from different suppliers. Initial slowness in the pace of vaccination and difficulties in achieving high vaccination coverage.

ICU: intensive care unit; SUS: Brazilian Unified National Health System.

Source: prepared by the authors, based on a set of sources and research material.

### Influences on national responses to COVID-19

The analysis of the three cases reveals the influence of different structural, institutional, political and societal factors on responses to COVID-19, with similarities and differences between countries.

Regarding structural factors, it is noteworthy that these three countries have upper-middle incomes and are historically characterized as States that invested in creating health systems and implementing healthcare and surveillance policies under different approaches, which could favor the response to a health crisis. On the other hand, they are characterized by marked social inequalities, which impact health.

Latin American countries suffered from global economic, scientific, and technological development asymmetries that affected the availability of financial resources and inputs to fight the pandemic, expressed in difficulties accessing equipment, tests, and immunization. In the case of vaccines, Brazil met some internal needs through the national public producers Fiocruz and Butantan. However, production was delayed due to dependence on active pharmaceutical ingredients (API) imports and technological transfer agreements. Argentina and Mexico faced some constraints in their cooperation strategy for binational production. The three countries competed with others to purchase vaccines from international companies, with Argentina being the most agile in this process and Brazil being the most resistant and slow, which generated criticism concerning the Federal Government.

Territorial inequalities and those between social groups influenced the health system’s responsiveness and the pandemic’s social and health impact by affecting health conditions, supply distribution, and access to services. Employment and social protection measures were insufficient to mitigate the crisis’s impact, especially on groups in situations of social vulnerability.

In addition to the structural dimension, institutional and political issues impacted national responses, with differences between countries. In Brazil’s case, the SUS and the previous institutional arrangements for federative coordination and social control would be favorable to combating COVID-19, but they were not properly taken into account. The SUS response was hampered by insufficient public funding and setbacks in health policies in recent years, aggravated by the denialism of the President and his power group, decision-making not based on scientific evidence, and little dialogue with subnational governments, experts and different social groups, widely expressed in the media and public statements by the various actors.

In the case of Argentina and Mexico, the health systems are more segmented, and the institutional mechanisms for federative health coordination are fragile. However, the political scenario was more favorable than in Brazil. The case of Argentina is distinguished by an initial response with solid action from the Presidency and a federative coordination effort by the Argentine Ministry of Health, with the support of provincial and municipal governments in the first year. The measures to restrict economic and social activities in favor of distancing were forceful, which was fundamental in containing the spread of COVID-19 and “flattening the curve of cases” in the first months of the pandemic in 2020. However, it was difficult to sustain these measures subsequently, given the drop in support from subnational governments and the population.

In Mexico, the response in the first year (2020) was hampered by the scenario of reforming a health system marked by high fragmentation and poor funding, by some hesitation on the part of the President regarding the seriousness of the crisis, and by more significant difficulties in national coordination. However, despite structural and institutional difficulties, there was political commitment and efforts to correct course of the struggle against the pandemic, including concerning immunization. Previous experience in dealing with epidemics in the country, such as H1N1 in 2009, allowed prevention, monitoring, and control of the disease to be organized.

Societal factors were also relevant, especially the efforts of universities and the scientific community in the three countries to produce technologies and knowledge to support health policies and private sector initiatives. Social and community movements contributed to minimizing the impacts of the crisis locally and among groups in vulnerable situations in the face of government failures. On the other hand, negative societal factors can be identified, such as the resistance of economic agents and the population to restrictive measures, such as closing shops and services, physical distancing, and the use of masks. Another harmful movement, more evident in Brazil, was the dissemination of fake news by denialist groups, with consequences such as vaccine hesitancy, especially regarding the vaccination of children.

Temporality is an important dimension in analysis of the factors that influenced the response, given the changes in the evolution of the pandemic and in the strategies for confronting it at each moment. In 2020, in the first year of the pandemic, control of COVID-19 relied on the coordination of measures to contain the spread of the virus, the response capacity of the health system (in surveillance, primary and hospital care), and measures to mitigate the economic, social and health effects of the crisis. The preconditions of health systems, the position of political leaders, and the capacity for dialogue and coordination were fundamental.

From 2021 onwards, vaccination will become decisive for controlling the disease, depending on the availability of effective vaccines, the capacity of the health system to vaccinate the country’s entire population in a timely and effective manner, and the population’s adherence to vaccination. These three elements are, in turn, influenced by asymmetries in the wealth and scientific, technological, and industrial development of countries, the characteristics of health systems, the political position of governments, and their relations with society, including information and communication.

Box 2 summarizes the favorable and unfavorable factors that influenced the national responses to the crisis.

**Box 2:** Factors that influenced the response of the studied countries to COVID-19: main favorable and unfavorable aspects, 2020 to 2022.

DIMENSION	FAVORABLE	UNFAVORABLE
Structural	Upper-middle-income countries State participation in shaping health systems and policies	Social inequalities Global asymmetries in resources and STI
Historical-institutional	Public health systems - SUS (in Brazil), existence of public providers, tradition, and previous experiences in public health and surveillance actions (in the three countries) Role of universities and public research institutions Some national capacity for the production of inputs and vaccines	Limits on investments in public systems; fragmentation/segmentation Little appreciation of primary health care; difficulties in integrating between primary health care and surveillance; low testing Limits in STI and dependence on imports
Political-conjunctural	National leadership efforts, with intergovernmental dialogue (Argentina) Subnational government initiatives (Brazil)	Difficulties in federative and intersectoral coordination and conflicts within and between governments Political decisions not based on scientific evidence and social dialogue (Brazil) Resistance from governments to the adoption of restrictive measures on economic and social activities
Societal	Commitment from public health organizations, scientific societies, and experts Mobilization of communities in some areas High adherence to adult vaccination	Low adherence to social distancing measures and the use of masks “Fake news” and, in some groups, hesitation to vaccinate children

STI: science, technology and innovation; SUS: Brazilian Unified National Health System.

Source: prepared by the authors, based on a set of sources and research material.

## Discussion

The study of Latin American federations suggested that the repercussions of COVID-19 in the countries were influenced by a combination of structural and institutional factors, such as State capacity, federative arrangements, and the configuration of the health system. Politics also played its role: the leadership of the national government and the more or less favorable orientation toward the coordination of actions, the implementation of scientifically based strategies, and social dialogue were relevant.

Regarding structural conditions, ECLAC highlights the factors that favored the spread of the disease and made it difficult to control in several nations in the region: adverse living and health conditions, the predominance of precarious employment relationships, insufficient urban and housing infrastructure, poor conditions of public transport, among others, associated with marked inequalities and weaknesses in social protection systems ^4^. Furthermore, structural conditions are decisive in the economic and social recovery capacity of countries in the face of the multidimensional crisis related to the pandemic ^4^ ^,^ ^34^.

Other studies highlight the more significant social impact of the pandemic on contexts of inequality and on groups in vulnerable situations, such as black people, Indigenous people, people with low incomes, and older people ^35^. Bambra et al. ^36^ indicate that the unequal effects of the pandemic between social groups are manifested in three plans: in mortality, which expresses socioeconomic and ethnic-racial inequalities; in lived experience, since poor people are less able to protect themselves from the disease; in impoverishment, given the drastic effects on lower-income workers, women, and young people. However, the authors stress that structural factors are insufficient to explain the pandemic’s unequal repercussions, as governments’ political choices influence the results.

This study made it possible to identify three aspects that differentiated the responses of the studied federations: (i) the density of governance and national coordination strategies between policy areas, between government/administration spheres, and in dialogue with society, considering political leadership and technical-scientific capacity; (ii) the articulation of pandemic containment and mitigation measures, i.e., the association of disease control actions with social protection, employment, and the economy; (iii) the response capacity of the health system, regarding speed, investments, and adequacy of health surveillance, diagnosis, health care and provision of equipment and supplies, including tests and vaccines.

Analyzing these aspects helps explain a more dramatic situation in Brazil and Mexico. The difficulties of federative coordination in these countries were accentuated and aggravated in the Brazilian case by the Federal Government’s position of denial and the intensification of federative, political-party conflicts and conflicts with scientific entities. The social protection and employment measures adopted in both countries were insufficient to meet the needs given the previous weaknesses of their economies and most of the population’s precarious working and living conditions. Concerning health systems, both Brazil and Mexico failed to prioritize the articulation between health surveillance and primary care in the response, with investments being directed mainly to the hospital network, which faced overload moments due to the insufficient and unequally distributed structure. Testing levels were low, making active surveillance and monitoring of the epidemiological situation difficult.

In Brazil, the existence of a public and universal system, the SUS, was essential but not sufficient to ensure an adequate response to the pandemic, given the previous limits in public financing and infrastructure, the unequal distribution of services, and the contradiction represented by the force of the private sector, subsidized by the State. Furthermore, the national situation was adverse to social policies and federative coordination. The national political situation was a decisive element, leading to tragic results in Brazil, a country that could have performed better, as other authors also pointed out, considering the national ^37^ and subnational ^38^ dimensions.

In Mexico, socioeconomic and territorial inequalities were aggravated by the difficulties of a segmented, fragmented and historically underfunded healthcare system, which was undergoing a reform process aiming at greater integration. Previous experience responding to the H1N1 emergency was essential for initiating health surveillance strategies ^39^. However, there was criticism from states and the scientific community regarding the President’s initial delay in acknowledging the seriousness of the pandemic and the concentration of power at the federal level, which was insufficient to ensure national coordination of actions and good results in controlling the pandemic ^40^.

In the case of Argentina, a federation characterized by greater decentralization, including the health sector, the initial response was marked by the Federal Government’s leadership in a “centralization and hyper-presidential” process ^41^. According to Cravacuore ^42^, coordination mechanisms in 2020 relied more on the President’s decision-making, with support from governors and mayors, than on stable institutional arrangements. Intergovernmental conflicts and population resistance to containment measures increased as the crisis prolonged. Furthermore, despite the greater availability of physicians and beds in Argentina compared with other Latin American countries, the health system’s highly segmented and fragmented nature made it difficult to respond to the health emergency.

## Conclusion

The study limits were the focus on national policies and the lack of interviews. Further studies are necessary to understand the diversity of subnational governments’ responses to COVID-19 in federative scenarios, the actions of civil society, and the perspectives of different actors. Analyzing the relationships between structural inequalities, other public policies, the health system’s response, and the different impacts of the pandemic on the territory and between social groups are topics that should be addressed in future investigation.

The study brought relevant lessons about Latin American countries’ challenges in what regards preparedness to respond to health emergencies. The first concerns the need to strengthen institutional mechanisms for national coordination of policies to face health emergencies, especially in scenarios of political-administrative decentralization, such as in federations. In addition to intergovernmental coordination, coordination between policy areas, public organizations, and societal organizations is essential.

The second lesson concerns the need to strengthen public health systems, including adequate financing, sufficient provision of services, availability and distribution of qualified professionals, and health supplies necessary for universal and accessible healthcare. Primary healthcare needs to be comprehensive and articulated with other services to ensure complex care, such as intensive care, which is generally concentrated and unequally accessed across the territory and between social groups.

The third lesson refers to the importance of investments in scientific and technological development in health in Latin American countries to reduce global asymmetries and guarantee the availability of health supplies, medicines, tests, and vaccines for the timely response to health emergencies.

The fourth lesson concerns the need to strengthen national social protection systems, expand labor rights, and implement comprehensive, universal, and focalized social policies based on a broad conception of citizenship and a commitment to reducing social inequalities.

The fifth lesson concerns State-society relations: it is essential to ensure social participation in public policies, whether during stable or crisis contexts. In health emergencies, dialogue with different organizations and social movements, including groups in social vulnerability, is necessary to ensure appropriate and effective policies.

Finally, strengthening the capacities of Nation-states is relevant but insufficient to respond to health emergencies of international importance, such as the COVID-19 pandemic. Promoting regional health integration between Latin American nations and cooperation with other countries in the Global South, such as those in Africa and the BRICS bloc (Brazil, Russia, India, China, and South Africa), is essential. Furthermore, in an asymmetric and unequal world, multilateral institutions must ensure global solidarity mechanisms, which have proven fragile in the current crisis.
