# Supplementary material for: The response to COVID-19 in Argentina, Brazil, and Mexico: challenges to national coordination of health policies
Source: Cad Saude Publica. 2024 Jul 29;40(6):e00055023. doi: 10.1590/0102-311XEN055023 (PMC11290836; doi:10.1590/0102-311XEN055023)
Supplement: Translation

# Translation: A resposta à COVID-19 na Argentina, no Brasil e no México: desafios à coordenação federativa das políticas de saúde

**DOI:** 10.1590/0102-311XPT055023

**Authors:** Cristiani Vieira Machado, Adelyne Maria Mendes Pereira, Carlos Machado de Freitas, Michele Souza e Souza, Sebastián Tobar, Suelen Carlos de Oliveira

**Affiliations:** 1 Escola Nacional de Saúde Pública Sergio Arouca, Fundação Oswaldo Cruz, Rio de Janeiro, Brasil.; 2 Instituto de Medicina Social, Universidade do Estado do Rio de Janeiro, Rio de Janeiro, Brasil.; 3 Centro de Relações Internacionais em Saúde, Fundação Oswaldo Cruz, Rio de Janeiro, Brasil.; 4 Universidade do Grande Rio, Mesquita, Brasil.

**Keywords:** Pandemias, COVID-19, Políticas de Saúde, Federalismo

## Abstract

Este artigo analisa o enfrentamento da COVID-19 em três federações latino-americanas: Argentina, Brasil e México. Realizou-se um estudo de casos múltiplos em perspectiva comparada, baseado em revisão bibliográfica, análise documental e de dados secundários, considerando: características dos países e do sistema de saúde, evolução da COVID-19, governança nacional, medidas de contenção e mitigação, resposta dos sistemas de saúde, condicionantes, aspectos positivos e limites das respostas. Os três países apresentavam sistemas de saúde distintos, porém marcados por financiamento insuficiente e desigualdades quando atingidos pela pandemia, e registraram alta mortalidade por COVID-19. As respostas nacionais foram influenciadas por condicionantes estruturais, institucionais e políticos. Na Argentina, a liderança nacional e acordos políticos intergovernamentais favoreceram a adoção inicial de medidas centralizadas de controle, que não se sustentaram. No Brasil, houve limites na coordenação e liderança nacional, relacionadas ao negacionismo do presidente e a conflitos federativos, políticos e com especialistas, apesar da existência de um sistema de saúde universal que têm comissões intergovernamentais e conselhos participativos, pouco acionados na pandemia. No México, dificuldades estruturais se associaram à relutância inicial do governo nacional em adotar medidas restritivas, limites na testagem e relativa lentidão na vacinação. Conclui-se que o enfrentamento de emergências sanitárias requer o fortalecimento dos sistemas públicos de saúde associados a mecanismos de coordenação federativa, intersetorial e com a sociedade civil, bem como mecanismos efetivos de solidariedade global.

## Introdução

A coordenação nacional de políticas de saúde envolve desafios em países federativos, em que o poder estatal é compartilhado por diferentes esferas de governo, envolvendo disputas por poder e recursos ^1^. Isso é complexo em nações extensas, heterogêneas e desiguais, como as federações latino-americanas da Argentina, do Brasil e do México.

Esses países, que juntos compreendem 67% do produto interno bruto e 59% da população da América Latina e Caribe ^2^, passaram, nas últimas décadas, por transformações no Estado e na sociedade, incluindo processos de democratização e descentralização político-administrativa. Entre as mudanças, assinalem-se reformas dos sistemas de saúde, de diferentes sentidos, com repercussões para a organização político-territorial, o direito e o acesso da população à saúde ^3^.

A partir de março de 2020, a COVID-19 atingiu duramente a região latino-americana, que passava por um cenário de crise econômica, exacerbando desigualdades em diversas dimensões ^4^. A pandemia revelou fragilidades das políticas sociais e de saúde nos países, expressas em insuficiente capacidade estatal para lidar com uma emergência sanitária complexa, fragmentação das políticas e limites de comunicação com a sociedade.

Os grupos em situação de vulnerabilidade social sofreram drasticamente os efeitos econômicos, sociais e sanitários da pandemia ^5^, diante das precárias condições de vida, de saúde e de inserção laboral, agravadas por sistemas de proteção social frágeis e investimentos insuficientes nos sistemas públicos de saúde.

O enfrentamento da COVID-19 exigia a articulação de estratégias como: medidas de distanciamento físico, isolamento ou quarentena, regulação de espaços públicos, ações de proteção individual e coletiva, reorganização do sistema de saúde, medidas econômicas e de proteção social, e iniciativas voltadas a distintos territórios e grupos sociais. A resposta dos países foi variada quanto à adoção de medidas de contenção e mitigação e à capacidade de coordenação entre esferas de governo, políticas públicas e com a sociedade, o que influenciou a efetividade das ações. Nas federações, tais processos foram moldados pela configuração político-territorial do Estado, poder e responsabilidades das esferas governamentais, características da descentralização e mecanismos de coordenação federativa, em geral e na saúde ^6^ ^,^ ^7^.

O estudo dessas três federações latino-americanas - Argentina, Brasil e México - é relevante, dada a magnitude dos efeitos da COVID-19 nesses países, que representam 4,9% da população mundial, mas responderam por 7% dos casos e 17% dos óbitos por COVID-19 confirmados no mundo até dezembro de 2022 ^8^. Além de combinarem, de forma contraditória, relevância econômica regional e marcantes desigualdades, tais nações enfrentam desafios de coordenação de políticas em cenários federativos marcados por fragmentação institucional e conflitos políticos.

Reconhecendo que a coordenação da resposta a crises sanitárias em federações apresenta especificidades ^6^ ^,^ ^7^ ^,^ ^9^, esta pesquisa objetivou identificar as principais características, os condicionantes, os elementos positivos e os limites nas respostas à pandemia de COVID-19 nessas nações latino-americanas. A partir dos seus resultados, busca-se extrair lições sobre os desafios dos sistemas públicos de saúde no enfrentamento de emergências sanitárias, em países marcados por desigualdades e dificuldades na coordenação de políticas públicas.

## Metodologia

Realizou-se um estudo de casos múltiplos em perspectiva comparada, apoiado em contribuições da abordagem histórico-comparativa das Ciências Sociais ^10^ e da literatura comparada sobre sistemas de saúde ^11^. Considerou-se, também, a bibliografia disponível sobre a resposta e resiliência dos sistemas de saúde diante da COVID-19 ^12^ ^,^ ^13^, para a identificação das dimensões relevantes para a análise.

Os países selecionados foram Argentina, Brasil e México, federações extensas, populosas e desiguais. O foco da pesquisa foi a condução nacional das políticas de enfrentamento da COVID-19, com ênfase na coordenação das estratégias, considerando os eixos de análise:

Contexto e características dos países e do sistema de saúde: indicadores socioeconômicos, demográficos e de saúde, configuração do sistema de saúde;

Situação epidemiológica da COVID-19: evolução da incidência e da mortalidade;

Governança nacional: partindo da definição de governança como padrão de relações entre atores estatais e não estatais, formais e informais, em ambientes institucionais ^14^, valorizou-se a coordenação federativa, entre áreas da política e com outros atores; liderança e comunicação com a sociedade;

Contenção e mitigação: controle de fronteiras, distanciamento físico, regulação de atividades comerciais e de lazer, medidas econômicas, proteção social e ao emprego;

Resposta dos sistemas de saúde à COVID-19 na vigilância e atenção à saúde: informações, vigilância ativa, testagem, articulação entre ações e serviços da atenção primária à hospitalar, vacinação;

Condicionantes, aspectos positivos e limites das respostas dos países.

O estudo se baseou em fontes secundárias. As características dos países e dos sistemas de saúde foram descritas com dados da Comissão Econômica para a América Latina e o Caribe (CEPAL) ^2^, do Banco Mundial ^15^, da Organização Mundial da Saúde (OMS) ^16^ e da Organização para a Cooperação e Desenvolvimento Econômico (OECD) ^17^, bases selecionadas por sua confiabilidade e disponibilidade de dados internacionais, complementadas por dados nacionais ^18^ ^,^ ^19^ ^,^ ^20^ ^,^ ^21^ ^,^ ^22^. A evolução da COVID-19, a testagem e a vacinação foram descritas com dados da base Our World in Data (Nosso Mundo em Dados) ^8^ e da Organização Pan-Americana da Saúde (OPAS) ^23^. Para caracterizar as respostas nacionais, diante do caráter recente e dinâmico da pandemia, além de revisão bibliográfica, as técnicas de pesquisa envolveram análise de sítios e documentos dos governos (planos, informes, relatórios, notas, atas), notas de sociedades científicas e profissionais e declarações de autoridades públicas, em matérias ou vídeos.

Os casos foram caracterizados e analisados em perspectiva comparada, buscando similaridades e diferenças nos diferentes eixos, nos condicionantes das políticas e nos aspectos positivos e limites da resposta à COVID-19 em cada país.

## Resultados

### Contexto dos países e características dos sistemas de saúde

As três federações latino-americanas são países populosos de renda média-alta, com importância econômica regional. Os processos nacionais de modernização industrial no século XX não alteraram sua inserção periférica no cenário mundial, nem desigualdades estruturais evidentes desde o período colonial. A pobreza, as desigualdades de renda e a informalidade no mercado laboral são mais acentuadas no Brasil e no México do que na Argentina. A expectativa de vida e a proporção de idosos são mais elevadas na Argentina, porém a taxa de mortalidade por doenças não transmissíveis e a prevalência de diabetes são mais altas no Brasil e no México, contribuindo para elevada carga de doenças. Quanto à estrutura do sistema de saúde, México e Argentina apresentam a maior oferta de médicos, e o Brasil, de enfermeiros. A Argentina tem maior disponibilidade de leitos, enquanto o México apresenta a menor oferta hospitalar, como mostra a Tabela 1.

**Tabela 1:** Indicadores demográficos, socioeconômicos, de saúde, de estrutura e financiamento e cobertura do sistema de saúde, antes da pandemia de COVID-19. Argentina, Brasil e México - 2019 ou último disponível.

Indicadores	Argentina	Brasil	México
Demográficos			
População total (milhões) (2019) *	44.938	211.782	125.085
População com 65 anos ou mais (% da população total) (2019) *	11,6	9,0	7,9
Socioeconômicos			
PIB *per capita* (USD) (2019) *	9.963,67	8.845,32	10.145,15
Pobreza (% da população) (2019) **	11,2	26,2	31,1
Razão do rendimento médio familiar *per capita* (quintil 5/quintil 1) (2019) **	8,5	19,1	11,9
Índice de Gini (2019) **	0,429	0,535	0,467
Empregos vulneráveis (% do total de empregos) (2019) *	22,7	28,3	27,2
Saúde			
Expectativa de vida (2019) ***	76,6	75,9	76,0
Taxa de mortalidade padronizada por doenças não transmissíveis (por 100 mil habitantes) (2019) ***	435,2	424,5	464,8
Probabilidade de morte entre 30-70 anos por DCV, câncer, diabetes ou DRC (2019) **	15,7	15,5	15,6
Prevalência de obesidade entre adultos (IMC ≥ 30, estimativa %) (2016) ***	28,5	22,3	28,4
Prevalência de diabetes (% da população de 20 a 79 anos) (2019) **	5,4	8,8	16,9
Estrutura do sistema de saúde			
Médicos (por 10 mil habitantes) (2019) ***	39,8	23,0	24,7
Enfermeiros (por 10 mil habitantes) (2019) ***	25,9	73,7	28,8
Leitos (por 10 mil habitantes) (2019) ***	49,9	20,9	9,9
Leitos de UTI (por 10 mil habitantes) (2019) ^#^	19,0	21,6	3,3
Financiamento do sistema de saúde			
Gasto em saúde (% do PIB) (2019) ***	10,0	9,6	5,4
Gasto público em saúde (% do PIB) (2019) ***	6,1	3,9	2,7
Gasto púbico em saúde *per capita* (USD PPC) (2019) ***	1,399	599	542
Gasto público em saúde (% do gasto público total) (2019) ***	16,1	9,2	10,3
Gasto privado em saúde (% do gasto em saúde) (2019) ***	39,2	59,1	50,8
Desembolso direto (% do gasto em saúde) (2019) ***	23,9	24,9	42,3
Cobertura do sistema de saúde (por tipo)			
Sistema público (não contributivo e de acesso gratuito, por meio de serviços estatais ou contratados) ^##^	100,0/34,8	100,0	36,5
Seguridade Social (contributiva, para trabalhadores) ^##^	62,7	NA	61,1
Setor privado de planos e seguros de saúde/medicina “pré-paga” ^##^	13,6	24,2	2,8

DCV: doenças cardiovasculares; DRC: doença renal crônica; IMC: índice de massa corporal; NA: não aplicável; PIB: produto interno bruto; PPC: paridade do poder de compra; UTI: unidade de tratamento intensivo.

Fonte: elaboração própria, com dados disponíveis nas bases:

* Banco Mundial ^15^;

** Comissão Econômica para a América Latina e o Caribe ^2^. Dados de pobreza, razão do rendimento e índice de Gini da Argentina são só para a população urbana; e os do México são referentes a 2018;

*** Organização Mundial da Saúde ^16^. Dados da Argentina relativos a enfermeiros são de 2017;

^#^ Para a Argentina, dados de 2019 do Sistema Integrado de Informação Sanitária, obtido de Gilardino et al. ^18^; para o Brasil, dados de janeiro de 2020 da Associação de Medicina Intensiva Brasileira ^19^; para o México, dados de 2017 da Organização para a Cooperação e Desenvolvimento Econômico/Banco Mundial ^17^;

^##^ Para a Argentina, dados do Ministério da Saúde referentes a 2019, disponíveis em Tobar ^20^; toda a população pode acessar os serviços públicos (100%), e 34,8% só tem acesso a eles. Para o Brasil, cujo sistema é público e universal (o Sistema Único de Saúde), dados de planos e seguros de saúde da Agência Nacional de Saúde Suplementar ^21^, referentes a 2019. Para o México, utilizaram-se os dados de “*derechohabiencia*” de 2020, do Instituto Nacional de Estatística e Geografia ^22^.

Nos três países, a organização das políticas de saúde na primeira metade do século XX ocorreu, de um lado, pela conformação de ações de saúde pública, voltadas ao controle de doenças infecciosas; por outro, pela assistência médica para trabalhadores formais, na lógica segmentada de seguro social. Porém, os processos de expansão de cobertura e configuração dos sistemas de saúde variaram no tempo, e as reformas setoriais a partir dos anos 1980 tiveram sentidos distintos ^3^ ^,^ ^24^.

Na Argentina, o modelo de seguro social se manteve na saúde por meio das Obras Sociais, organizações vinculadas aos sindicatos responsáveis pela gestão de recursos e atenção à saúde, inicialmente por categorias. As reformas dos anos 1980 e 1990 introduziram mecanismos de mercado que fragilizaram as bases corporativas, como a possibilidade de livre escolha das Obras Sociais pelos trabalhadores e a contratação de empresas de saúde para a prestação de serviços. Isso favoreceu a expansão do setor de medicina pré-paga, regulado por lei a partir de 2011. O subsistema de saúde pública, da atenção primária à hospitalar, é de responsabilidade das províncias e municípios, com configurações diversas. Há alta descentralização, autonomia das províncias na legislação, organização e fiscalização dos serviços, sob escassa regulação e financiamento pelo Ministério da Saúde. O Conselho Federal de Saúde (COFESA) é uma instância de articulação entre a autoridade nacional e as autoridades provinciais de saúde, cuja institucionalidade e relevância variaram no tempo ^25^. As dificuldades de coordenação federativa em saúde expressam características do federalismo argentino ^26^. O sistema nacional de saúde é altamente segmentado (por grupos de beneficiários) e fragmentado (organizacionalmente), pelo caráter dos subsistemas das Obras Sociais, provinciais e setor privado, com interconexões complexas entre eles ^27^.

No Brasil, a reforma sanitária dos anos 1980 levou à criação do Sistema Único de Saúde (SUS) em 1988, público e universal, cuja implementação favoreceu o aumento da oferta, cobertura e acesso aos serviços públicos. Houve transformações no modelo de atenção, como o fortalecimento de políticas orientadas pela integralidade e a expansão da atenção primária, por meio da Estratégia Saúde da Família. Diante da descentralização político-administrativa, com ênfase nos municípios, o Ministério da Saúde manteve poder regulatório por meio de normas e transferências financeiras federais. As mudanças incluíram arranjos de participação social e de coordenação federativa na saúde, como as comissões intergestores nacional e estaduais, que conferiram institucionalidade a processos compartilhados de construção e acompanhamento das políticas ^26^ ^,^ ^28^. Como limites, registre-se a insuficiência do financiamento em saúde e a persistência do setor privado, subsidiado pelo Estado (desde os anos 1960), expressos no baixo gasto público e alta proporção de gastos privados em saúde, contraditórios com o SUS ^29^.

No México, nos anos 1980 e 1990, o sistema de saúde passou por mudanças no bojo de reformas do Estado que enfatizavam a liberalização econômica, privatização, redução de gastos públicos e descentralização ^30^. Houve reforma da previdência e tentativas de restringir a assistência médica ligada ao Instituto Mexicano de Seguridade Social (IMSS) e ao Instituto de Seguridade e Serviços Sociais dos Trabalhadores do Estado (ISSSTE), obstruídas pelos sindicatos. A partir dos anos 2000, implantou-se o Seguro Popular de Saúde (SPS) voltado aos pobres não cobertos pela Seguridade, que acessavam serviços públicos de saúde descentralizados. Nos anos subsequentes, observou-se aumento da população cadastrada pelo SPS, sob controvérsias, pelo caráter delimitado dos serviços incluídos no pacote básico e pelo fato do cadastramento não garantir acesso ^31^. Em 2018, o novo governo extinguiu o SPS e criou o Instituto de Saúde para o Bem-Estar, que estava em implantação inicial quando o país foi atingido pela COVID-19. São características do sistema mexicano: fragmentação organizacional, segmentação de cobertura, baixos gastos públicos e instabilidade relacionada às sucessivas reformas.

Em que pesem as diferenças entre os países, antes da pandemia todos apresentavam problemas sociais como pobreza, desigualdades de renda e alta informalidade laboral, que dificultariam o enfrentamento da crise. Seus sistemas de saúde expressavam limites e contradições, como insuficiente gasto público e alta proporção de gastos privados em saúde, no caso do Brasil principalmente por meio de planos e seguros de saúde (apesar do SUS) e no México por desembolso direto das famílias (Tabela 1).

### Situação epidemiológica da COVID-19

Os três países foram duramente atingidos pela COVID-19 a partir de 2020, alcançando, até o final de 2022, altos níveis de mortalidade acumulada em comparação com outras federações do mundo. A Tabela 2 mostra que, no período, o Brasil teve a maior taxa de mortalidade e o México as maiores estimativas de letalidade e de excesso de mortalidade (em relação à série histórica de óbitos por todas as causas em anos anteriores), em comparação com outras federações do mundo. A incidência nos três países possivelmente está subestimada em virtude da baixa testagem, o que pode levar à subnotificação de casos e, em menor escala, de óbitos, afetando a letalidade.

**Tabela 2:** Indicadores relativos à COVID-19 na Argentina, Brasil, México e em outras federações selecionadas - até 2022.

País	Incidência acumulada *	Mortalidade acumulada **	Excesso de mortalidade (%) ***	Letalidade (%) ^#^			Testes (por 1.000 habitantes) ^##^	Cobertura vacinal (%) ^###^	Doses de reforço (por 100 habitantes) pessoas ^§^
				2020	2021	2022			
Argentina	217.338,36	2.859,22	19,24	2,92	2,12	1,32	773,09	76,10	60,45
Brasil	167.775,53	3.217,37	20,44	2,56	2,78	1,92	330,91	78,29	48,22
México	56.826,32	2.598,52	29,53	9,90	7,58	4,57	117,38	62,70	41,69
Canadá	116.407,83	1.259,18	6,31	2,73	1,52	1,08	1.538,60	81,77	57,16
Estados Unidos	292.706,07	3.192,46	14,04	1,81	1,57	1,09	2.483,63	67,28	37,64
Alemanha	446.707,54	1.987,98	6,32	2,83	1,68	0,45	1.357,56	76,03	68,33
África do Sul	67.590,07	1.712,49	17,97	2,67	2,66	2,53	393,41	31,76	5,71
Índia	31.525,51	374,47	ND	1,45	1,38	1,19	551,94	64,19	2,95
Rússia	150.395,92	2.718,36	24,29	1,80	2,93	1,81	1.928,73	51,36	10,09
Austrália	408.521,74	680,59	4,45	3,25	0,65	0,17	2.487,17	82,68	61,53

ND: não disponível.

Fonte: Mathieu et al. ^8^.

* Incidência acumulada: casos confirmados/milhão de habitantes, até 25/Dez/2022;

** Mortalidade acumulada: óbitos confirmados/milhão de habitantes, até 25/Dez/2022;

*** Excesso de mortalidade (%): a diferença percentual entre o número acumulado de mortes por todas as causas desde janeiro de 2020 e o número de mortes projetado para o período, com base em anos anteriores. Dados de dezembro de 2022 para todos os países, com exceção da Argentina, cujos dados são de dezembro de 2021;

^#^ Letalidade (%): relação entre os óbitos confirmados por COVID-19 e os casos confirmados de COVID-19. O indicador não é uma medida precisa do risco de morte, por ser prejudicado pela subnotificação de casos leves. Para cada ano, foram obtidos os últimos dados disponíveis na última semana de dezembro;

^##^ Testes: número cumulativo de testes por 1.000 habitantes, com variações da unidade de medida: testes realizados (Argentina, Brasil, Canadá, Estados Unidos, Alemanha, Rússia e Austrália); pessoas testadas (México e África do Sul); amostras testadas (Índia). Dados de 06/Mar/2022 para a Alemanha e de 11/Mar/2022 para os demais países;

^###^ Cobertura vacinal: % da população com esquema vacinal inicial completo. Dados de 16/Jun/2022 para a Austrália, e de 24/Jun/2022 para os demais. O mês de junho foi escolhido para fins de comparabilidade;

^§^ Doses de reforço: número de doses de reforço por 100 habitantes. Dados de 24/Jun/2022.

A Figura 1 mostra sucessivas ondas de mortalidade nos países estudados. Observa-se início mais tardio e lento da primeira onda na Argentina, em 2020, favorecido pelas medidas rigorosas de distanciamento social e vigilância no primeiro ano da pandemia, coordenadas pelo governo federal, em articulação com as províncias. Posteriormente a “fadiga social” levou ao relaxamento das medidas ^20^ e houve crescimento acentuado da mortalidade, influenciado também pela circulação de diferentes variantes do vírus.

**Figura 1:** Evolução de novos óbitos diários por COVID-19 por milhão de habitantes e percentual da população com esquema vacinal inicial completo. Argentina, Brasil e México - março/2020 a junho/2022.

Fonte: elaboração própria. Dados obtidos de Mathieu et al. ^8^.

A segunda onda teve início primeiro no México, mas o Brasil e a Argentina atingiram picos altos de mortalidade por COVID-19. No caso brasileiro, essa onda elevada se prolongou do final de 2020 a 2021. A vacinação começou gradualmente no início de 2021, e em meados do ano se observou um efeito mais contundente do aumento da cobertura vacinal no controle da mortalidade na Argentina e no Brasil. No México, onde a vacinação foi mais lenta, ocorreu uma terceira onda ainda nesse ano. No início de 2022, os países passaram por nova onda, dessa vez relacionada à variante ômicron, que levou a altíssimas incidências, com mortalidade menor do que a associada às ondas anteriores, devido à proteção coletiva relacionada ao avanço da vacinação.

### Governança nacional, medidas de contenção e resposta dos sistemas de saúde

A governança nacional e a capacidade de resposta à COVID-19 variaram entre os países, influenciadas pelos distintos arranjos federativos e posição dos governos, quanto à orientação política, liderança e capacidade de diálogo com os especialistas e outros grupos sociais.

A Argentina tem o arranjo federativo mais descentralizado dos três, com alta autonomia das províncias na saúde. Em 2020, houve centralização de medidas pelo Governo Federal e mecanismos de coordenação federativa envolveram reuniões e acordos entre autoridades políticas e sanitárias. Essa articulação foi importante para o controle inicial da COVID-19, incluindo restrições legais à mobilidade e a atividades econômicas e sociais para assegurar o distanciamento físico. Houve investimentos principalmente nos hospitais, com expansão de leitos.

Dado o prolongamento da crise de COVID-19, as estratégias de articulação esmoreceram, diante do caráter muito descentralizado da federação argentina, da competição política e da escassez de arranjos institucionais mais sólidos de cooperação intergovernamental. Na sociedade, em um cenário de instabilidade econômica, a resistência às medidas de contenção cresceu, e a “fadiga social” levou à redução da adesão da população ao distanciamento físico. O início precoce da vacinação em dezembro de 2020 e sua expansão em 2021, mediante compra de diferentes fornecedores, foi crucial para conter os casos graves, internações e óbitos por COVID-19. Também houve um arranjo de produção regional, em parceria com o México, que teve atrasos e resultados limitados. A capacidade nacional de pesquisa, a atuação de universidades e parcerias público-privadas permitiram a produção local e fornecimento de máscaras, respiradores e outros insumos estratégicos, com indução e apoio federal ^32^.

O Brasil se caracteriza pela descentralização político-administrativa em saúde com ênfase nos municípios, porém com poder do Governo Federal na indução de políticas e papel variado dos estados. A existência das comissões intergestores em saúde nos âmbitos nacional e estadual é importante para a coordenação federativa no SUS ^26^, e a atuação de conselhos de saúde participativos nas três esferas de governo é relevante para o controle social. Porém, esses canais não foram valorizados na resposta à COVID-19, observando-se conflitos intergovernamentais e limites de articulação entre governos e com a sociedade. Isso foi agravado pela posição do presidente da República de negacionismo, recomendação de medidas sem base científica e exacerbação de conflitos com oponentes. A conformação de um comitê nacional de emergência não foi suficiente para compensar os problemas da liderança e coordenação. Houve fragilização do Ministério da Saúde como autoridade sanitária nacional, com três trocas de ministros entre 2020 e 2022 e atrasos na implementação de medidas relevantes.

Em um governo marcado por restrições ao gasto público federal e retrocessos na área social, a limitada coordenação intersetorial prejudicou a articulação entre políticas de saúde, de proteção social e econômicas para lidar com as múltiplas dimensões da crise. Como aspectos positivos, destaca-se a existência do SUS e da extensa rede de atenção primária à saúde, cujo potencial de contribuir no enfrentamento da emergência foi pouco valorizado ^33^. A vacinação ilustrou contradições na política nacional. De um lado, o país tinha um Programa Nacional de Imunizações (PNI) abrangente, e a capacidade de produção nacional de vacinas por duas instituições públicas - Fundação Oswaldo Cruz - Fiocruz (federal) e Instituto Butantan (estadual) -, que forneceram mais de metade das doses administradas até janeiro de 2023 ^23^ - incluindo acordos de transferência de tecnologia para a produção nacional de vacinas. De outro, a posição da presidência prejudicou a vacinação, com atrasos em decisões de aquisição e declarações negativas sobre vacinas, fragilizando o PNI, que desfrutava de alta credibilidade internacional e legitimidade junto à população.

No México, a federação mais centralizada das três, a liderança e a coordenação do Governo Federal seriam decisivas para a resposta, diante dos baixos gastos públicos, da fragmentação do sistema de saúde e do processo de reforma do sistema de saúde em curso. Contudo, a hesitação inicial na adoção das medidas necessárias de contenção prejudicou o controle da propagação da COVID-19. A organização da resposta baseou-se em experiências prévias de estruturação da vigilância em saúde para o enfrentamento de emergências sanitárias. Porém, os limites de testagem e a lentidão na vacinação foram fragilidades, já que dependiam da aquisição de diferentes fornecedores. Tais limites, associados à segmentação do sistema de saúde (expressa nas diferenças entre os subsistemas em termos do desenvolvimento de diretrizes, protocolos, infraestrutura e insumos), ao persistente subfinanciamento público e às desigualdades estruturais nas condições de saúde e acesso, contribuíram para alta mortalidade por COVID-19 e excesso de mortalidade no período.

O Quadro 1 resume as características das respostas dos três países à pandemia.

**Quadro 1:** Principais características da resposta dos países à COVID-19. Argentina, Brasil e México, 2020 a 2022.

PAÍS	PRINCIPAIS CARACTERÍSTICAS DA RESPOSTA DOS PAÍSES
Argentina	Governança e coordenação nacional: esforço de governança suprasetorial entre áreas do governo e diálogo com especialistas; mecanismos pactuados de coordenação federativa em saúde, porém com frágil institucionalidade; Medidas de contenção e mitigação: inicialmente estratégias de fechamento de fronteiras (com dificuldades), estratégias de distanciamento obrigatórias, medidas econômicas e de proteção social. Depois, “fadiga social” e resistências às medidas - novas ondas; Resposta do sistema de saúde: sistema de saúde segmentado, mas com oferta hospitalar robusta. Investimentos do Governo Federal (aumento de leitos de UTI, respiradores, contratações). Inovações e busca de articulação entre setores. Limitado papel da atenção primária à saúde. Início da vacinação em dezembro de 2020 e boa disponibilidade de vacinas, mediante a compra de fornecedores variados. Relevância do alcance de alta cobertura vacinal a partir de 2021 para conter os casos graves e mortalidade.
Brasil	Governança e coordenação nacional: limites na liderança nacional e coordenação federativa e intersetorial; escassa consideração de evidências científicas e participação da sociedade; Medidas de contenção e mitigação: inicialmente estratégias de fechamento de fronteiras (com dificuldades), tensões entre Governo Federal e especialistas e variação entre estados e municípios quanto às medidas de distanciamento; medidas econômicas e de proteção social insuficientes; Resposta do sistema de saúde: relevância do SUS, mas dificuldades por desigualdades e baixos investimentos; ênfase na atenção hospitalar (expansão de leitos), limitada articulação entre setores público e privado, pouca valorização da atenção primária à saúde na resposta; em 2020, escassa testagem, dificuldades na importação de insumos e equipamentos. Relevância da produção nacional de testes e vacinas (responsável por mais da metade das doses ministradas até 2022), com transferência tecnológica. Papel decisivo da vacinação a partir de meados de 2021, com boa cobertura vacinal de adultos com o esquema inicial básico. Porém, dificuldades de alta cobertura das doses de reforço e entre crianças.
México	Governança e coordenação nacional: esforço de diretrizes nacionais, especialmente para vigilância, mas dificuldades na coordenação federativa junto aos estados; Medidas de contenção e mitigação: demora inicial na adoção de medidas de distanciamento físico; depois programa nacional, alcance limitado. Insuficiência e baixa coordenação de medidas de proteção à economia, ao emprego e de proteção social; Resposta do sistema de saúde: sistema de saúde segmentado e fragmentado (em reforma), com desigualdades de infraestrutura, limites de financiamento público e disponibilidade de equipamentos e insumos. Estratégias de vigilância com base na experiência prévia com emergências sanitárias. Ênfase na atenção hospitalar e testagem restrita a casos graves. Dependência da compra de vacinas de fornecedores variados. Lentidão inicial no ritmo da vacinação e dificuldades no alcance de altas coberturas vacinais.

SUS: Sistema Único de Saúde; UTI: unidade de terapia intensiva.

Fonte: elaboração própria, a partir do conjunto de fontes e material de pesquisa.

### Condicionantes da resposta nacional dos países à COVID-19

A análise dos três casos revela a influência de diferentes condicionantes sobre as respostas à COVID-19, de caráter estrutural, institucional, político e societal, com semelhanças e diferenças entre os países.

No que concerne aos fatores estruturais, destaca-se o fato de esses três países apresentarem renda média-alta e se caracterizarem, historicamente, como Estados que investiram na conformação de sistemas e na implementação de políticas de atenção e vigilância à saúde, sob variados formatos, o que poderia favorecer a resposta a uma crise sanitária. Por outro lado, são marcados por desigualdades sociais acentuadas, que repercutem na saúde.

Os países latino-americanos sofreram com as assimetrias globais no desenvolvimento econômico, científico e tecnológico que afetaram a disponibilidade de recursos financeiros e de insumos para o enfrentamento da pandemia, o que se expressou em dificuldades de acesso a equipamentos, a testes e a vacinas. No caso das vacinas, o Brasil, por meio dos produtores nacionais públicos Fiocruz e Butantan, conseguiu atender parte das necessidades internas, mas a produção atrasou pela dependência de importação de ingrediente farmacêutico ativo (IFA) e de acordos de transferência tecnológica. Argentina e México encontraram limites em sua estratégia de cooperação para a produção binacional. Os três países competiram com outros pela compra de imunizantes de empresas internacionais, a Argentina sendo mais ágil nesse processo, e o Brasil mais resistente e lento, o que gerou críticas ao Governo Federal.

As desigualdades territoriais e entre grupos sociais influenciaram a capacidade de resposta do sistema de saúde e o impacto social e sanitário da pandemia, ao afetarem as condições de saúde, a distribuição da oferta e o acesso aos serviços. As medidas de proteção ao emprego e sociais foram insuficientes para atenuar o impacto da crise, especialmente sobre os grupos em situação de vulnerabilidade social.

Além da dimensão estrutural, questões institucionais e políticas repercutiram nas respostas nacionais, com diferenças entre os países. No caso do Brasil, a existência do SUS, bem como os arranjos institucionais prévios de coordenação federativa e de controle social, seriam favoráveis ao enfrentamento da COVID-19, mas não foram devidamente acionados. A resposta do SUS foi prejudicada pelo insuficiente financiamento público e retrocessos nas políticas de saúde nos últimos anos, agravadas pela posição do presidente e seu grupo de poder, de negacionismo, tomada de decisões não fundamentadas na ciência, e escasso diálogo com governos subnacionais, especialistas e os diversos grupos sociais, amplamente expressos na mídia, em posicionamentos e declarações públicas dos diversos atores.

Já no caso da Argentina e do México, os sistemas de saúde são mais segmentados e os mecanismos institucionais de coordenação federativa em saúde são frágeis. No entanto, o cenário político foi mais favorável do que no Brasil. O caso da Argentina se diferencia por uma resposta inicial com forte atuação da Presidência da República e esforço de coordenação federativa pelo Ministério da Saúde, com adesão de governos provinciais e municipais no primeiro ano. As medidas de restrição às atividades econômicas e sociais em prol do distanciamento foram contundentes, o que foi fundamental para conter a propagação da COVID-19 e “achatar a curva” nos primeiros meses da pandemia, em 2020, embora com dificuldade de sustentação posterior dessas medidas, da adesão dos governos subnacionais e da população.

No México, a resposta no primeiro ano (2020) foi dificultada pelo cenário de reforma de um sistema de saúde marcado por alta fragmentação e baixos investimentos, por certa hesitação do presidente quanto à gravidade da crise e por maiores dificuldades de coordenação nacional. No entanto, apesar de dificuldades estruturais e institucionais, houve comprometimento político e esforços de correção de rumos, inclusive no que concerne à vacinação. A experiência prévia com o enfrentamento de epidemias no país, como a da H1N1 em 2009, possibilitou que ações de prevenção, monitoramento e controle da doença fossem organizadas.

Condicionantes societais também foram relevantes. Destacam-se os esforços das universidades e da comunidade científica dos três países em produzir tecnologias e conhecimento para apoiar as políticas de saúde e as iniciativas do setor privado. Movimentos sociais e comunitários contribuíram para minimizar os impactos da crise localmente e junto a grupos em situação de vulnerabilidade, diante de falhas dos governos. Por outro lado, foram elementos negativos a dificuldade de adesão dos agentes econômicos e da população a medidas de restrição, como: fechamento de comércio e serviços; distanciamento físico e uso de máscaras; e a disseminação de notícias falsas, mais grave no Brasil, articuladas por grupos negacionistas, com consequências como hesitação vacinal, especialmente quanto à vacinação de crianças.

Uma dimensão importante na análise dos condicionantes da resposta é a temporalidade, dadas as mudanças na evolução da pandemia e nas estratégias para seu enfrentamento em cada momento. Em 2020, no primeiro ano da pandemia, o controle da COVID-19 dependeu da articulação de medidas de contenção da propagação do vírus, da capacidade de resposta do sistema de saúde (na vigilância, na atenção primária e hospitalar), e de medidas de mitigação dos efeitos econômicos, sociais e sanitários da crise. As condições prévias dos sistemas de saúde, a posição das lideranças políticas e a capacidade de diálogo e coordenação foram fundamentais.

A partir de 2021, a vacinação passa a ser decisiva para o controle da doença, dependendo da disponibilidade de vacinas eficazes, da capacidade do sistema de saúde vacinar toda a população do país de forma oportuna e efetiva e da adesão da população à vacinação. Esses três elementos são, por sua vez, influenciados pelas assimetrias na riqueza e no desenvolvimento científico, tecnológico e industrial dos países, pelas características dos sistemas de saúde, pela posição política dos governos e suas relações com a sociedade, inclusive no plano da informação e da comunicação.

O Quadro 2 resume os aspectos favoráveis e desfavoráveis relativos aos diferentes grupos de condicionantes da resposta à crise.

**Quadro 2:** Condicionantes da resposta dos países estudados à COVID-19: principais aspectos favoráveis e desfavoráveis, 2020 a 2022.

CONDICIONANTES	FAVORÁVEIS	DESFAVORÁVEIS
Estrutural	Países de renda média-alta Participação do Estado na conformação de sistemas e políticas de saúde	Desigualdades sociais Assimetrias globais nos recursos e em C&TI
Históricos-institucionais	Sistemas públicos de saúde - SUS (no Brasil), existência de prestadores públicos, tradição e experiências prévias em ações de saúde pública e de vigilância (nos três países) Papel das universidades e instituições de pesquisa públicas Alguma capacidade nacional de produção de insumos e de vacinas	Limites de investimentos nos sistemas públicos; fragmentação/segmentação Pouca valorização da atenção primária à saúde; dificuldades de integração atenção primária à saúde-vigilância; baixa testagem Limites na CT&I e dependência de importações
Político-conjunturais	Esforços de liderança nacional, com diálogo intergovernamental (Argentina) Iniciativas de governos subnacionais (Brasil)	Dificuldades na coordenação federativa e intersetorial e conflitos intra e entre governos Decisões políticas sem base em evidências científicas e no diálogo social (Brasil) Resistência de governos à adoção de medidas restritivas às atividades econômicas e sociais
Societais	Compromisso de organizações de saúde pública, de sociedades científicas e de especialistas Mobilização das comunidades em algumas áreas Alta adesão à vacinação de adultos	Baixa adesão às medidas de distanciamento social e ao uso de máscaras *Fake news* e, em alguns grupos, hesitação à vacinação de crianças

CT&I: ciência, tecnologia e inovação; SUS: Sistema Único de Saúde.

Fonte: elaboração própria, a partir do conjunto de fontes e material de pesquisa.

## Discussão

O estudo das federações latino-americanas sugeriu que as repercussões da COVID-19 nos países expressaram a combinação de fatores estruturais com questões institucionais, como a capacidade do Estado, os arranjos federativos e a configuração do sistema de saúde. A política também importou; foram relevantes a liderança do governo nacional e a orientação mais ou menos favorável à coordenação de ações, à implementação de estratégias cientificamente embasadas e ao diálogo social.

Quanto às condições estruturais, a CEPAL destaca como fatores que favoreceram a propagação da doença e dificultaram o seu controle em várias nações da região: condições de vida e de saúde adversas; predomínio de vínculos de trabalho precários; infraestrutura urbana e de moradia insuficientes; más condições dos transportes públicos; entre outras, associadas às marcantes desigualdades e fragilidades dos sistemas de proteção social ^4^. Além disso, condições estruturais são decisivas na capacidade de recuperação econômica e social dos países, diante da crise multidimensional relacionada à pandemia ^4^ ^,^ ^34^.

Outros estudos destacam o maior impacto social da pandemia em contextos de desigualdades e em grupos em situação de vulnerabilidade, como pessoas negras, indígenas, pobres e idosos ^35^. Bambra et al. ^36^ sinalizam que efeitos desiguais da pandemia entre grupos sociais se manifestam em três planos: na mortalidade, que expressa desigualdades socioeconômicas e étnico-raciais; na experiência vivida, visto que pessoas pobres têm menos condições de se proteger da doença; e no empobrecimento, dados os efeitos drásticos sobre trabalhadores de menor renda, mulheres e jovens. Porém, as autoras alertam que fatores estruturais não são suficientes para explicar as repercussões desiguais da pandemia, pois as escolhas políticas dos governos influenciam os resultados.

Esta pesquisa permitiu identificar três aspectos que diferenciaram as respostas das federações estudadas: (1) a densidade das estratégias de governança e de coordenação nacional, entre áreas da política, entre esferas de governo e administração e no diálogo com a sociedade, considerando liderança política e capacidade técnico-científica; (2) a articulação das medidas de contenção e mitigação da pandemia, ou seja, a associação de ações de controle da doença com as de proteção social, ao emprego e à economia; (3) a capacidade de resposta do sistema de saúde, no que concerne à rapidez, investimentos e adequação de ações de vigilância, diagnóstico, assistência e provisão de equipamentos e insumos, incluindo testes e vacinas.

A análise desses aspectos ajuda a explicar uma situação mais dramática no Brasil e no México. As dificuldades de coordenação federativa nesses países foram acentuadas, agravadas no caso brasileiro pela posição do Governo Federal de negacionismo e de acirramento dos conflitos federativos, político-partidários e com entidades científicas. As medidas de proteção social e ao emprego adotadas nos dois países foram insuficientes para dar conta das necessidades diante das fragilidades prévias de suas economias e da precariedade das condições de trabalho e de vida da maior parte da população. No que concerne aos sistemas de saúde, ambos não conseguiram priorizar a articulação entre vigilância em saúde e atenção primária na resposta, sendo os investimentos direcionados principalmente para a rede hospitalar, que passou por momentos de sobrecarga diante da estrutura insuficiente e desigualmente distribuída. Os níveis de testagem foram baixos, dificultando a vigilância ativa e o monitoramento da situação epidemiológica.

No Brasil, a existência de um sistema público e universal, o SUS, foi importante, mas não suficiente para assegurar uma resposta adequada, em face dos limites prévios no financiamento público e na infraestrutura, da distribuição desigual de serviços e da contradição representada pela força do setor privado, subsidiado pelo Estado. Além disso, a conjuntura nacional foi adversa às políticas sociais e à coordenação federativa. A política foi um elemento decisivo, levando a resultados trágicos em um país que teria um potencial de resposta bem melhor, o que foi apontado também por outros autores, considerando as dimensões nacional ^37^ e subnacional ^38^.

Já no México, se somaram às desigualdades socioeconômicas e territoriais as dificuldades de um sistema de saúde segmentado, fragmentado e historicamente subfinanciado, que estava passando por um processo recente de reforma, com vistas à maior integração. A experiência prévia de resposta à emergência de H1N1 foi importante para a organização inicial das estratégias de vigilância em saúde ^39^. Porém, houve críticas dos estados e da comunidade científica à demora inicial do presidente em admitir a gravidade da pandemia e à concentração de poder na esfera federal, que foi insuficiente para assegurar a coordenação nacional de ações e bons resultados no controle da pandemia ^40^.

No caso da Argentina, federação caracterizada por maior descentralização, inclusive na saúde, a resposta inicial foi marcada pela liderança do Governo Federal, em um processo de “centralização e hiperpresidencialismo” ^41^. Conforme Cravacuore ^42^, os mecanismos de coordenação em 2020 dependeram mais da liderança do presidente, com apoio de governadores e prefeitos, do que de arranjos institucionais estáveis. Com o prolongamento da crise, aumentaram os conflitos intergovernamentais e a resistência da população às medidas de contenção. Além disso, apesar da maior disponibilidade de estrutura de médicos e leitos na Argentina em comparação a outros países latino-americanos, o caráter altamente segmentado e fragmentado do sistema de saúde dificultou a resposta à emergência sanitária.

### Considerações finais

Esta pesquisa teve como limites o foco nas políticas nacionais e a não realização de entrevistas. Outros estudos são necessários para compreender a diversidade de respostas dos governos subnacionais à COVID-19 em cenários federativos, a atuação da sociedade civil e as perspectivas dos diferentes atores. A análise das relações entre desigualdades estruturais, outras políticas públicas, resposta do sistema de saúde e diferentes impactos da pandemia no território e entre grupos sociais também requer a realização de outras pesquisas.

O estudo apresentou lições relevantes sobre os desafios dos países latino-americanos de preparação de respostas às emergências sanitárias. A primeira concerne à necessidade de fortalecer os mecanismos institucionais de coordenação nacional das políticas para enfrentar emergências sanitárias, especialmente em cenários de descentralização político-administrativa, como nas federações. Além da coordenação intergovernamental, é fundamental a articulação entre áreas da política, dos órgãos públicos e das organizações societais.

A segunda lição diz respeito à necessidade de fortalecer sistemas públicos de saúde, incluindo: financiamento adequado, oferta suficiente de serviços, disponibilidade e distribuição de profissionais qualificados e de insumos necessários para a atenção universal e gratuita à saúde. A atenção primária em saúde precisa ser abrangente e articulada aos demais níveis de atenção, para assegurar o atendimento até níveis complexos, como a terapia intensiva, em geral concentrada e de acesso desigual no território e entre grupos sociais.

A terceira lição se refere à importância de investimentos no desenvolvimento científico e tecnológico em saúde nos países latino-americanos para reduzir assimetrias globais e garantir a disponibilidade de insumos, medicamentos, testes e vacinas para a resposta oportuna a emergências sanitárias.

A quarta se relaciona à necessidade de fortalecer os sistemas nacionais de proteção social, com expansão de direitos trabalhistas e políticas sociais abrangentes, universais e focalizadas, baseadas em uma concepção ampla de cidadania e no compromisso de redução das desigualdades sociais.

A quinta lição se situa na esfera das relações Estado-sociedade: é essencial assegurar a participação social nas políticas públicas, em tempos de estabilidade ou de crise. Em emergências sanitárias, é necessário o diálogo com diferentes organizações e movimentos da sociedade, incluindo os grupos em situação de vulnerabilidade social, para que as políticas sejam adequadas e efetivas.

Por fim, o fortalecimento das capacidades dos Estados Nacionais é relevante, mas insuficiente para responder às emergências sanitárias de importância internacional, como a pandemia de COVID-19. É fundamental promover a integração regional em saúde entre nações da América Latina e a cooperação com outros países do Sul Global, como os da África e do bloco Brasil, Rússia, Índia, China, África do Sul (BRICS). Ademais, em um mundo assimétrico e desigual, as instituições multilaterais precisam assegurar mecanismos de solidariedade global, que se mostraram frágeis na crise atual.
